# Endovascular Fenestration in Aortic Type‐A Dissection With Hepatic Malperfusion Syndrome: A Case Report

**DOI:** 10.1002/ccr3.70347

**Published:** 2025-03-24

**Authors:** Daniel Weiss, Kai Jannusch, Lena Wilms, Hannan Dalyanoglu, Tim Ullrich, Gerald Antoch, Peter Minko, Farid Ziayee

**Affiliations:** ^1^ Department of Diagnostic and Interventional Radiology Medical Faculty and University Hospital Düsseldorf, Heinrich‐Heine‐University Düsseldorf Düsseldorf Germany; ^2^ Department of Cardiovascular Surgery Medical Faculty and University Hospital Düsseldorf, Heinrich‐Heine‐University Düsseldorf Düsseldorf Germany; ^3^ CARID Cardiovascular Research Institute Düsseldorf, University Hospital Düsseldorf, Heinrich‐Heine‐University Düsseldorf Germany

**Keywords:** cardiothoracic surgery, cardiovascular disorders, emergency medicine, radiology & imaging, vascular surgery

## Abstract

Acute type‐A aortic dissection with malperfusion syndromes represents challenging cases and should always be treated on an interdisciplinary basis, whereby interventional radiologic therapy can be a successful procedure, especially in patients who are not fit for surgery.

**Abbreviations**
CT‐A: computertomography angiography
DSA: digital subtraction angiography
GD: gastroduodenal
OCS: OUTBACK Elite Re‐entry Catheter
SIM: Simmons diagnostic catheter
SMA: superior mesenteric artery

## 1 Introduction

Acute type‐A aortic (DeBakey I, 80% of cases) dissection comes along with high rates of mortality in untreated patients and even in patients undergoing surgery up to 18% [1]. One of the primary concerns is the collapse of the true lumen due to increased pressure in the false lumen, leading to malperfusion of affected organs [2, 3]. Furthermore, surgical therapy is the gold standard of treatment; however, endovascular treatment alone or in combination with surgery may be considered. Especially if true lumen collapse has evolved after surgical treatment or if the patient is unfit for (re‐) operation, endovascular techniques should be evaluated on an interdisciplinary basis [4, 5].

## 2 Case History/Examination

### 2.1 Initial Presentation and First Surgery

**FIGURE 1:**
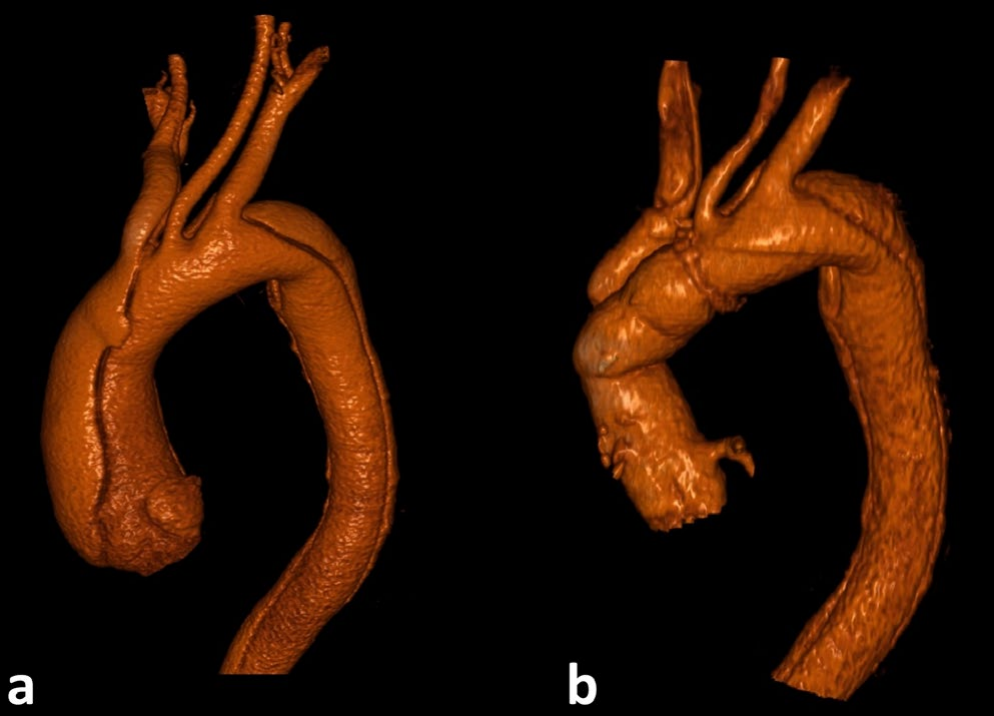
Pre‐ and postoperative 3D reconstructions of the thoracic aorta (a) Preoperative 3D reconstruction of the thoracic aorta showing an aortic type‐A dissection (DeBakey I) and an aneurysm of the ascending aorta with a diameter of 52 mm. Additionally, the involvement of the supra‐aortic branches was observed. (b) Postoperative 3D reconstruction of the thoracic aorta after a David procedure and partial aortic arch replacement (Gelweave Valsalva graft, 28 mm, Terumo, Vascutek GmbH, Germany).

A 48‐year‐old male patient presented to the emergency department of an external hospital with sudden severe back pain. Computed tomography angiography (CT‐A) revealed an aortic type‐A (DeBakey I) dissection, an aneurysm of the ascending aorta with a diameter of 52 mm and a known closure of the celiac trunk with retrograde opacification via the superior mesenteric artery (SMA). Given the urgency of the case, the patient was promptly transferred to the local cardiovascular department for immediate surgery. He underwent an immediate open surgical approach, including a David Procedure and partial aortic arch replacement (Figure 1).

### 2.2 Differential Diagnosis

#### 2.2.1 Postoperative Clinical Course and Imaging

**FIGURE 2:**
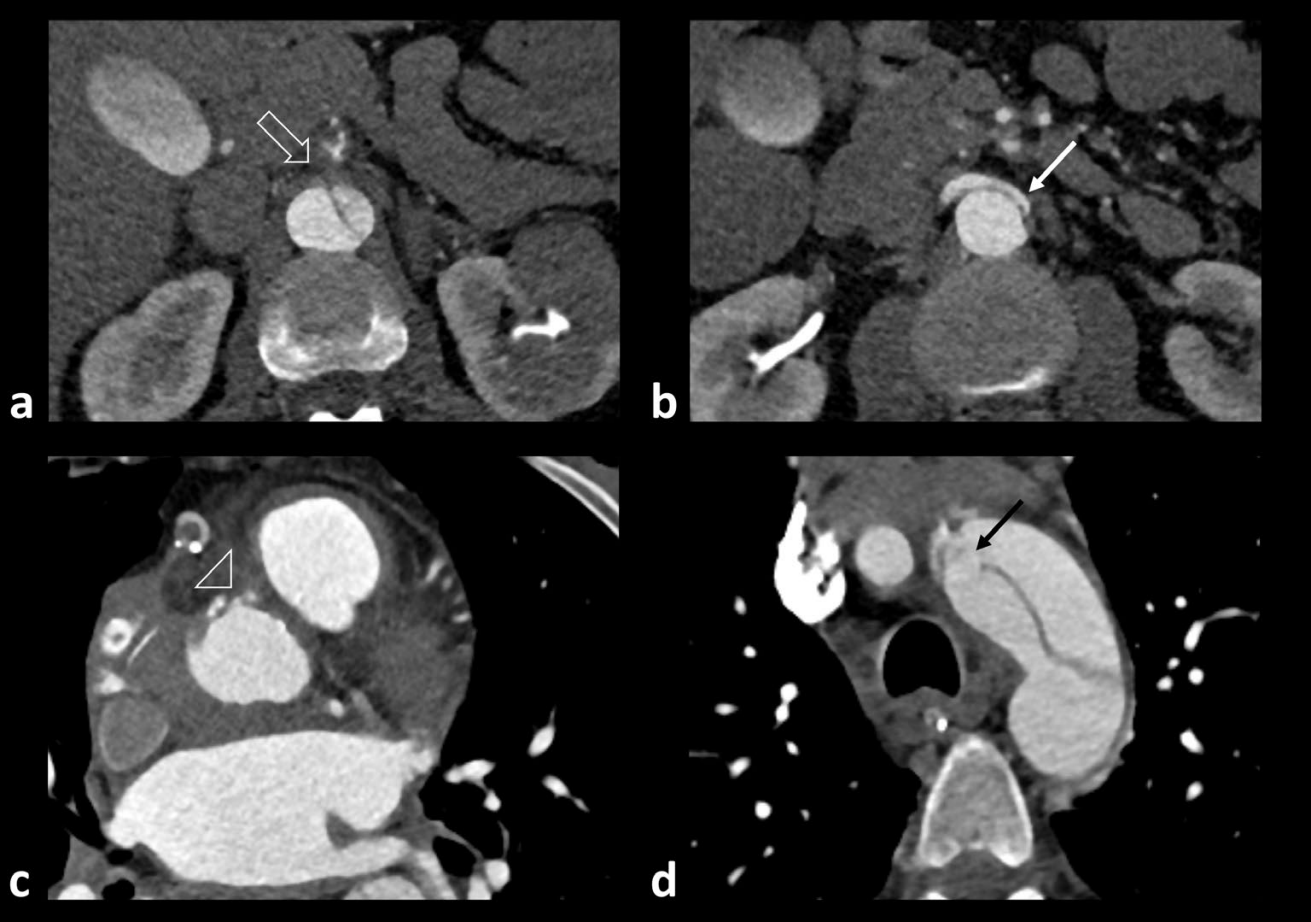
Postoperative findings in type‐A dissection with right coronary compression and aortic re‐entry. (a) Axial abdominal computed tomography angiography (CT‐A) after surgical treatment indicated compression of the right coronary artery by absorbable hemostyptic agent (white arrowhead). (b, c) Furthermore, it revealed a wide re‐entry of the aortic arch (black arrow) and a small re‐entry infrarenal (white arrow). (d) The chronic occlusion of the celiac trunk (white bold arrow) which was previously compensated via gastroduodenal collaterals but is currently accompanied by distinct hepatic malperfusion (not shown).

Initially, the postoperative course was uneventful. However, a sudden increase in CK‐MB was observed, prompting a conventional coronary angiography (Figure 2). This revealed a remarkable narrowing of the right coronary artery's ostium. To further investigate the cause of this narrowing and assess the status of the dissection, a CT‐A of the complete aorta was performed.

### 2.3 Thoracic Findings

Imaging identified the cause of the coronary narrowing as a compression of the right coronary artery by an absorbable hemostatic agent (Figure 2a), along with a wide re‐entry of the proximal aortic arch (Figure 2b,c).

### 2.4 Abdominal Findings

The celiac trunk had a chronic occlusion with collateral opacification via the gastroduodenal (GD) artery (Figure 2d). However, the current CT‐A indicated a distinct malperfusion of the liver combined with an increase in liver retention parameters. The underlying cause was a long‐distance collapse of the true lumen, extending down to the common iliac arteries.

### 2.5 Interdisciplinary Therapeutic Considerations

In principle, an urgent open thoracoabdominal repair with SMA and right renal reimplantation would be the gold standard in this situation [6, 7]. However, due to the patient's critical condition, he was deemed inoperable for an open surgical procedure. As a result, a multidisciplinary team opted for an endovascular treatment as a salvage strategy. The goal was to enhance perfusion via the SMA and subsequently improve collateral circulation to the liver. However, success in such an intervention can only be achieved through appropriate multidisciplinary collaboration, including the involvement of a major aortic service: an endovascular, longitudinal fenestration of the dissection membrane.

### 2.6 First Endovascular Treatment

**FIGURE 3:**
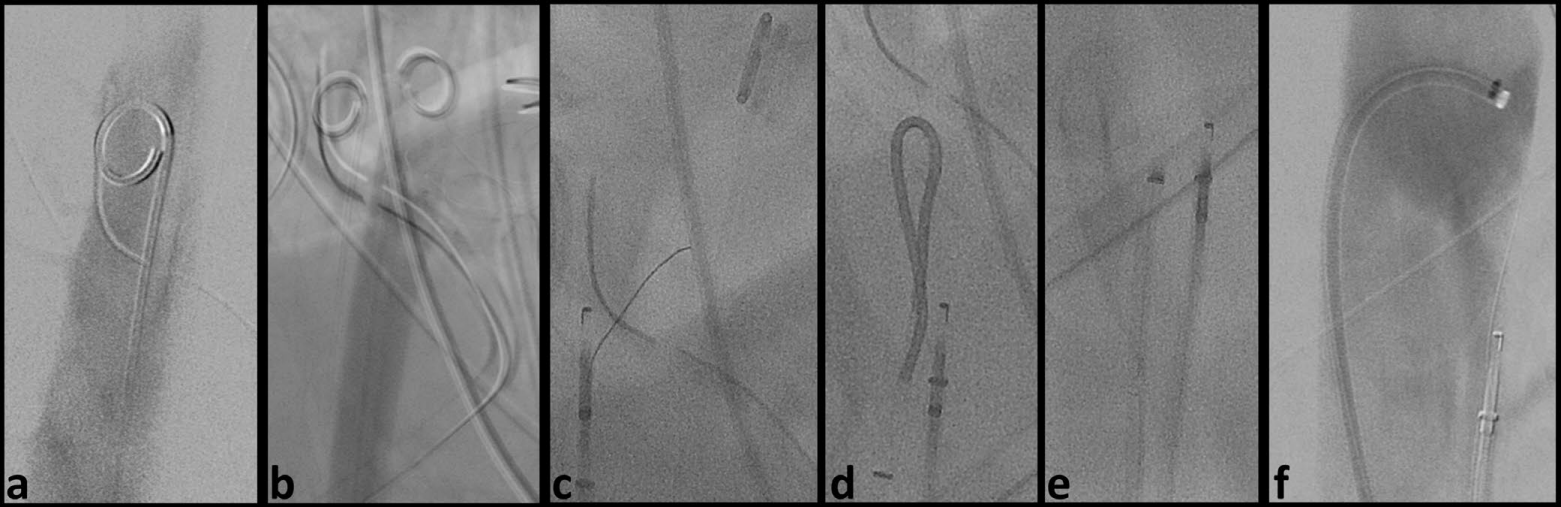
Endovascular longitudinal fenestration of abdominal dissection membrane using the body‐floss technique. Retrograde access over both common femoral arteries was established. (a, b) True lumen was probed from the left and false lumen from the right side with a 4F pigtail diagnostic catheter, and digital subtraction angiography (DSA) was performed (not shown) to confirm correct position. (c) Perforation of the dissection membrane with a stiff guide wire was not possible; therefore, perforation was performed using a 6F OUTBACK Elite Re‐entry Catheter (OCS, CORDIS, Cardinal Health, USA). (d) Successful perforation was confirmed by DSA (not shown) via a 5F SIM diagnostic catheter. (e) Subsequently, a microwire was introduced via the outback catheter system and captured with a 6F snare catheter, andboth, OCS, with retracted needle, and snare catheter, were dragged caudally in a “body‐floss” technique. (f) Confirmation of biluminal opacification was performed via DSA through a 6F diagnostic catheter.

The intervention was performed under general anesthesia due to the patient's unstable condition. Retrograde access was obtained via both common femoral arteries (Figure 3).

#### 2.6.1 Probing the True and False Vessel Lumen

The true lumen was probed from the left and the false lumen from the right side with a 4F pigtail diagnostic catheter. Digital subtraction angiography (DSA) confirmed the correct positioning (Figure 3a,b).

#### 2.6.2 Membrane Perforation

Initial attempts to perforate the dissection membrane using a stiff guidewire were unsuccessful. Consequently, a more invasive method with a 6F OUTBACK Elite Re‐entry Catheter (OCS, CORDIS, Cardinal Health, USA) was performed. The true lumen was considered too wide to afford enough radial force to perforate the membrane, so perforation was performed via the false lumen (Figure 3c). Confirmation of successful perforation was performed by DSA via a 5F Simmons (SIM) diagnostic catheter (Figure 3d).

#### 2.6.3 Membrane Slitting

Slitting with the OCS is risky, as an injury to the aortic wall can occur quickly. Thus, a microwire was introduced via the OCS and captured with a 6F snare system via true lumen. Both OCS, with retracted needle, and snare catheter were dragged caudally from thoracic vertebral body 11 to lumbar vertebral body 1 (Figure 3e). This was intended to create an equalization of flow conditions. Confirmation of biluminal opacification was performed via DSA (Figure 3f).

Postinterventional cone beam computed tomography showed improved opacification of collateralization between SMA and celiac trunk.

## 3 Conclusion and Results (Outcome and Follow‐Up)

### 3.1 Postinterventional Control Imaging

A follow‐up CT‐A was performed 12‐h post‐intervention, revealing significantly improved liver perfusion. Correspondingly, laboratory findings showed normalization of liver retention parameters. In the further course, liver and renal retention parameters increased, and hemodialysis was indicated. In control CT‐A, the true lumen at the level of the right renal ostium was almost completely occluded by the dissection membrane.

### 3.2 Second Endovascular Treatment

**FIGURE 4:**
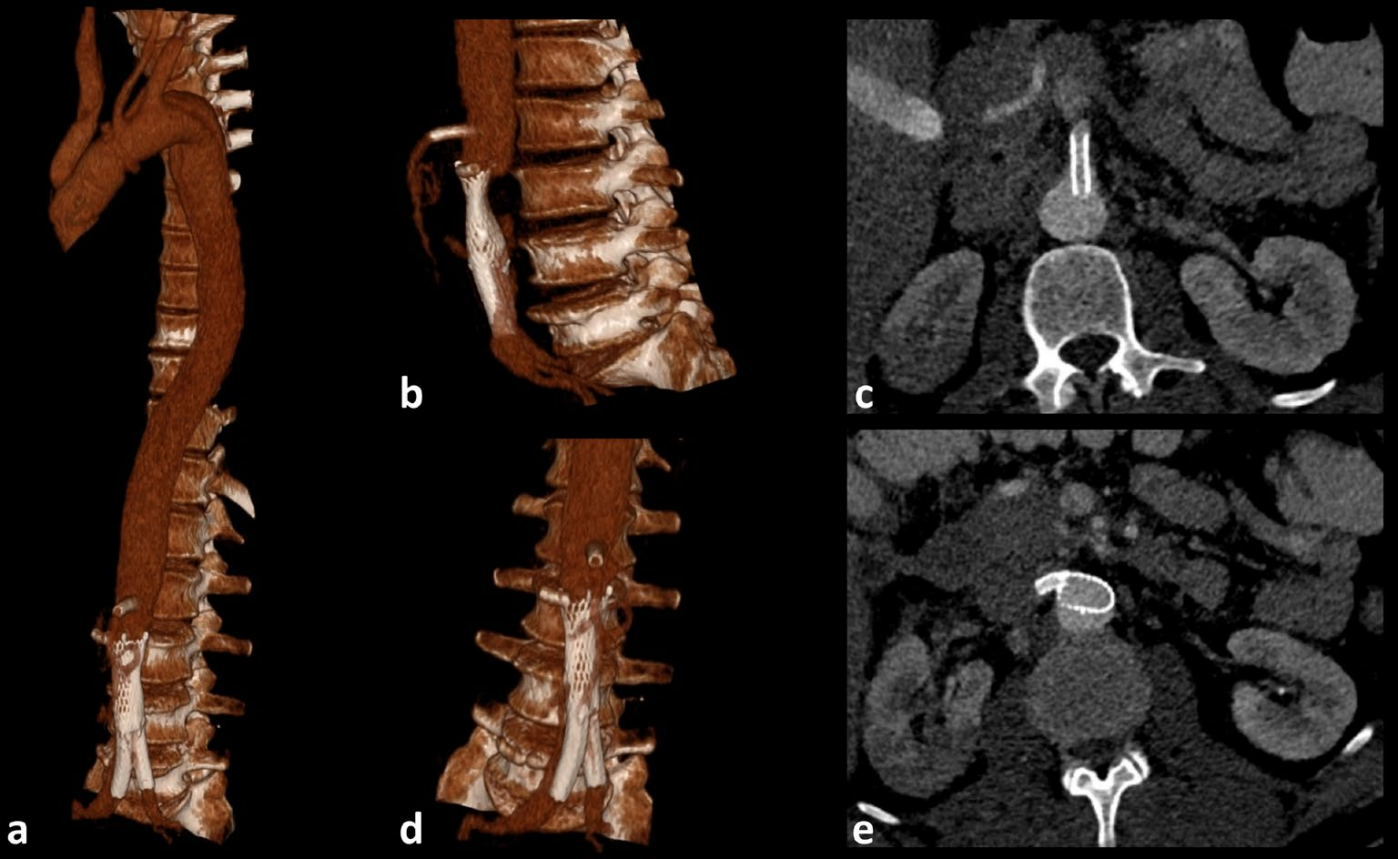
Final outcome of type‐A aortic dissection repair (a, b, d) 3D reconstructions showing the final results of surgical and interventional treatment for the aortic type‐A dissection. The procedures included a David procedure, partial aortic arch replacement, stenting of the superior mesenteric artery (SMA), stenting of the right renal artery, and implantation of two iliac stent‐grafts with remaining stenosis of the infrarenal aorta. (c, e) Axial abdominal computed tomography angiography demonstrating good opacification of the stents in the SMA (c) and the right renal artery (e). Additionally, partial infarction of the right kidney due to the initial dissection is visible.

A second intervention was performed using the same membrane slitting technique, extending from the infrarenal aorta to the aortic bifurcation (Figure 4). Additional procedures included:

Furthermore, a stenting of the SMA (6 × 24 mm balloon‐expandable Hippocampus Renal RX Stent System 0.014″; Medtronic, Minneapolis, MN, USA), a stenting of the right renal artery (6 × 15 mm balloon‐expandable Hippocampus Renal RX Stent System 0.014″; Medtronic, Minneapolis, MN, USA), an implantation of an infrarenal aortic stent (22 × 60 mm Sinus‐XL stent; OptiMed, Ettlingen, Germany), and the implantation of two iliacal stent‐grafts in y‐technique (9 × 57 mm BeGraft stent‐grafts; Bentley InnoMed GmbH, Hechingen, Germany) due to renal and lower extremity hypoperfusion were performed (Figure 4).

### 3.3 Post‐Interventional Course

Cardiac function was monitored using laboratory tests and echocardiography, with additional assessments conducted through multiple CT scans. Since cardiac function improved over time, no further intervention was required for the narrowing of the right coronary artery. Hemodialysis was continued, and there was a gradual improvement in the patient's condition. After approximately 3 weeks, the patient could be discharged from the intensive care unit to the normal ward and, after two further weeks, to follow‐up rehabilitation.

## 4 Discussion

Fenestration of a dissection membrane during aortic dissection as a therapeutic option in abdominal malperfusion syndromes was commonly performed in an open surgical approach in the last decades [6, 7, 8]. Endovascular fenestration of the dissection in the presence of existing malperfusion of abdominal organs is feasible for type‐B dissections. However, it is only considered as an alternative emergency intervention in cases of type‐A (DeBakey I) dissections when surgical intervention is impossible due to the patient's condition [4, 9].

In this report we demonstrated the performance of longitudinal fenestration in a patient with an acute type‐A dissection as a salvage intervention. Close interdisciplinary cooperation and a well‐defined overall treatment concept were crucial for managing such critical patients. Prior to this, appropriate diagnostics were employed to specify the vascular treatment target. In this case, malperfusion of the liver was indicated by CT‐A, which was exacerbated by chronic occlusion of the celiac trunk.

Angiographic biluminal visualization of the true and false lumen is a prerequisite for further procedure. In principle, a more careful puncture of the dissection membrane with a stiff wire could be attempted subsequently. However, success may depend on the thickness of the dissection membrane and the forces that can be applied to the wire: while thick membranes are often inherently more challenging to cut through, thinner walls are frequently more pliable. Furthermore, preinterventionally, it must be clearly assessed whether and, if so, through which lumen the probing of visceral vessel branches can be performed. In our case, however, this was not successful, so we resorted to a re‐entry system as an off‐label use, suitable for recanalization in the context of an occluded peripheral vessel. The subsequent “body‐floss” technique—that is, pulling the wire down from the false and true lumen using both catheters proved to be a very effective procedure [10]. After the initial endovascular treatment, we observed a recurrence of the patient's condition deterioration due to the progression of the dissection. Therefore, a repeat intervention was necessary. Nevertheless, especially in critically unstable patients, therapy should be symptom‐oriented, with the condition re‐evaluated to avoid overtreatment.

This technique may facilitate secondary stent‐graft placement by converting a two‐lumen into a single aortic lumen [10, 11, 12]. In this case, one could opt for either the implantation of a stent or a stent‐graft to treat limb ischemia. To ensure better coverage of the dissection membrane, we chose the implantation of a stent‐graft in this instance.

Nevertheless, the process is also accompanied by disadvantages. If the intervention is unsuccessful, other therapeutic options within the scope of an endovascular or surgical approach need to be evaluated, and the loss of time could adversely affect the affected organ. Additionally, the dissecting membrane may partially shift caudally and displace corresponding vascular outlets located here, usually the iliac vessels, and lead, for example, to limb ischemia in this scenario [10]. Therefore, close monitoring of the patient and proper interdisciplinary communication are crucial.

In individual cases, when surgical treatment is not possible, endovascular fenestration of the dissection membrane in acute type‐A (DeBakey I) aortic dissections may be considered. In this instance, it was an effective and safe treatment option. Successful outcomes depend on close interdisciplinary collaboration, a well‐planned therapeutic concept, and meticulous monitoring to avoid complications and achieve favorable results.

## Author Contributions

**Daniel Weiss:** conceptualization, data curation, formal analysis, investigation, methodology, project administration, validation, visualization, writing – original draft, writing – review and editing. **Kai Jannusch:** conceptualization, data curation, validation, writing – review and editing. **Lena Wilms:** conceptualization, data curation, validation, writing – review and editing. **Hannan Dalyanoglu:** conceptualization, data curation, validation, writing – review and editing. **Tim Ullrich:** conceptualization, data curation, validation, writing – original draft. **Gerald Antoch:** conceptualization, data curation, validation, writing – review and editing. **Peter Minko:** conceptualization, data curation, validation, writing – review and editing. **Farid Ziayee:** conceptualization, data curation, investigation, methodology, project administration, supervision, validation, writing – original draft, writing – review and editing.

## Ethics Statement

This case report was conducted in accordance with the Declaration of Helsinki. The Institutional Review Board of our hospital did not require ethics approval in this case report.

## Consent

Written informed consent was obtained from the patient to publish this report in accordance with the journal's patient consent policy.

## Conflicts of Interest

The authors declare no conflicts of interest.

## Data Availability

The data that support the findings of this study are available from the corresponding author upon reasonable request.
